# Supplementary material for: Factors associated with early pregnancy anemia in rural Sri Lanka: Does being ‘under care’ iron out socioeconomic disparities?
Source: PLoS One. 2022 Oct 6;17(10):e0274642. doi: 10.1371/journal.pone.0274642 (PMC9536542; doi:10.1371/journal.pone.0274642)
Supplement: Attachment — Submitted filename: plos one.docx [file pone.0274642.s002.docx]

**Factors associated with early pregnancy anemia in rural Sri Lanka:** **Does being 'under care' iron out socioeconomic disparities?**

Gayani Shashikala Amarasinghe^1^, Thilini Chanchala Agampodi^1^, Vasana Mendis^2^, Suneth Buddhika Agampodi^1,3*^

^1^ Department of Community Medicine, Faculty of Medicine and Allied Sciences, Rajarata University of Sri Lanka, Saliyapura, Sri Lanka

^2^ Department of Pathology, Faculty of Medicine and Allied Sciences, Rajarata University of Sri Lanka, Saliyapura, Sri Lanka

^3^ Department of Internal Medicine, Division of Infectious Diseases, Yale School of Medicine, New Haven, CT, USA

* Corresponding author

E-mail: suneth.agampodi@yale.edu

**Abstract**

Despite multiple interventions across the lifecycle, anaemia in pregnancy remains a challenge. Identifying associated factors will enable recognition of gaps in current preventive strategies, enabling refining them for better anaemia control. All first-trimester pregnant women registered for antenatal care in the Anuradhapura district between July 2019? and September 2019 were invited to participate in the study. Interviewer-administered and self-completed questionnaires and full blood count were used. Hierarchical logistic regression was performed to identify associated factors. The Period of Gestation (PoG), maternal age and parity were added in block one. Breast feeding within the past year, history of anaemia, BMI, having a vehicle at home, having the last pregnancy five or more years back and having used contraceptive injections and intrauterine contraceptive devices (IUCD) for one year or more were in block two. Total of 3128 participants, 451 (14.4%) were anaemic. According to the model (Chi-square =139.3, p<0.001, n = 2692) the odds of being anaemic increased with the PoG (OR =1.07, 95% CI=1.01-1.13).While controlling for PoG, age and parity, history of anaemia (OR=3.22, 95%CI=2.51-4.13), being underweight (OR=1.64, 95%CI=1.24-2.18), having the last pregnancy five or more years back (OR=1.57,95%CI=1.15-2.15) and having used IUCD for one year or more (OR=1.63, 95%CI=1.16-2.30) increased the odds of anaemia. Breast feeding during the last year (OR=0.66, 95%CI=0.49-0.90) and having used contraceptive injections for one year or more (OR=0.61,95%CI=0.45-0.83) reduced the risk of anaemia. Proxy indicators of being in frequent contact with the national family health programme has a protective effect over socioeconomic disparities in preventing early pregnancy anaemia. Maintaining the continuum of care through the lifecycle, especially through optimization of pre and interpregnancy care provision, is essential.

Key words?

**Introduction**

Anaemia results from inadequate red cells or haemoglobin concentration to maintain the optimum oxygen supply for tissues(1). Anaemia in pregnant women could result in significant transgenerational morbidity, mortality and productivity loss. Pregnant women are especially vulnerable to anaemia for several reasons, including physiological changes, socioeconomic or cultural restrictions to achieve optimal nutrition and increased nutritional demand during pregnancy. In fact, 40% of pregnant women worldwide are anaemic(2). A significant proportion of them, especially those identified as anaemic during the early stages of pregnancy, would have already had anaemia or marginal haemoglobin values prior to pregnancy. Therefore prevention of anaemia in pregnancy is essentially linked with anaemia control in reproductive-age women.

Global progress in reducing anaemia prevalence among pregnant women has been plodding (3). Why a significant proportion of pregnant women would remain vulnerable to anaemia despite multiple interventions at different stages of the lifecycle is a question that needs answers. Identifying factors associated with anaemia is as crucial as identifying the underlying etiologies for anaemia when answering this question. Studies have shown that healthcare utilization during pregnancy, including the reception of antenatal care, nutrient supplements and deworming, and utilization of general healthcare services such as family planning, significantly reduced anaemia in pregnant or non-pregnant reproductive age females(3,4). Individual dietary patterns such as increased meat, fish and egg consumption and community-level food fortification programs were also associated with anaemia prevention(3). Odds of being anaemic decreased with increased maternal age, increased gap between pregnancies and lower parity. Across geographical regions, underweight women had an increased risk of anaemia compared to normal or overweight women. Education level, wealth, employment status, hygiene and sanitary facilities were also associated with anaemia(3).

Sri Lanka is a lower middle income country providing well structured family health services free of charge at the point of delivery with an island wide coverage(5). Public health provision has a strong orientation towards the continumum of care across life cycle(6). Despite countrie’s continuous efforts, close to a third of pregnant women are anaemic and prevalanece of anaemia among first trimester women is 18.4%(6). Early studies in Sri Lanka have shown that anaemia was more prevalent among pregnant women with a high parity (7). However, perhaps due to the success of family planning programme, this association was not reflected in later studies (8). Pregnant women at either extremities of reproductive age reported a higher prevalence of anaemia and iron deficiency(8). Iron folate supplementation prior to the booking visit did not prevent anemia(8). Iron folate supplementation during pregnancy which is incoperated in the national maternal care package reduced the risk of anaemia (5)(9). Dietary habits such as milk, tea and red meat consumption failed to show an association with anaemia in pregnancy, but higher consumption of green leaves and eggs were associated with a lower risk for anemia(10). An association between anaemia and the income level also has been shown(10). Studies on non-pregnant reproductive age females have shown that anemia was not associated with economic status or dietary habits such as vegetarianism, consuming tea within an hour of a meal, and consuming meat, fish and eggs (11). Age, parity, and educational level were associated with anameia (12,13). Local studies reporting data on factors associated with anaemia have employed heterogeneous study samples impairing the ability to generate an in-depth understanding of the determinants of maternal anaemia, which would be invaluable to design further anaemia control strategies. Therefore, we conducted this study to identify the factors associated with early pregnancy anaemia in a large community-based sample of pregnant women from a single geographical area in the country. Identifying associated factors enabled recognizing gaps in the current preventive strategies, alerting stakeholders to refine them to achieve better outcomes.

**Materials and Methods**

We carried out this descriptive cross-sectional study as a component of the baseline assessment of the Rajarata pregnancy cohort(14). All the first trimester (Period of gestation less than 13 weeks) pregnant women registered in the pregnant mothers' registers of public health midwives in Anuradhapura district between July and September 2019 were invited to participate in the study. Anuradhapura is the geographically largest of the 25 administrative districts in the country. For the participant recruitment, special clinics were conducted weekly or fortnightly in each of the 22 medical officers of health (MOH) areas in the district. Anaemia prevalence in the recruited sample of first trimester women (n=3127) was 14.4% (n=451), and iron deficiency, minor haemoglobinopathies and vitamin B12 deficiency were identified as the major etiological contributors to anaemia (15).

An interviewer-administered questionnaire was used to obtain demographic and health-related data. Menstrual blood loss was assessed using a pictogram. Health records, including the current and previous pregnancy records and high-performance liquid chromatography (HPLC) for thalassemia screening, were checked to verify the obtained information. Socioeconomic data and data on dietary habits were obtained using self-completed questionnaires. Anthropometric measurements were obtained following a standard protocol. A full blood count was performed in all the participants from a public health research laboratory with external and internal quality control methods (16). Anaemia was defined as a haemoglobin level less than 11 g/dl. Minor haemoglobinopathies were identified based on the red cell index (microcytic anaemia with a high red cell count) which showed 100% positive predictive value in the HPLC tested subsample(15). A detailed account of the study is presented elsewhere(17).

Data were analyzed using SPSS version 22. Hierarchical logistic regression was performed to identify factors associated with anaemia among first-trimester pregnant women.

Ethical clearance

This study was approved by the ethics review commmiitte of Faculty of Medicine and Allied Sciences, Rajarata University of Sri Lanka. Informed written conscent was obtained from all participants.

**Results**

Out of the 3127 first trimester pregnant women recruited, 215 (7.5%) were teenage and majority were ethnic Sinhalese (n=2730, 87.1%), followed by Moor/Malay (n=367, 11.7%) and Tamil (n=39,1.2%), respectively. The majority (N=1873, 60.3%) were educated up to the GCE ordinary level. Of the participants, 1071 (34.3%) were prim gravid, and 1158 (37.1%) had one child. Monthly family income varied between 2000 rupees (9.9 USD) to 0.5 million rupees (2469 USD). The median was 40,000 rupees (197.6 USD). Underweight, normal, overweight and obesity were seen in 504 (16.6%), 993 (32.7%), 485 (16.0%) and 1053 (34.7%) respectively. The mean period of gestation (PoG) was eight weeks, and hemoglobin distribution according to the gestation revealed a reducing trend after 10 weeks PoGs (figure 1).

**Fig 1 Haemoglobin distribution of first trimester pregnant women by the period of gestation**

There were 561 participants who had a history of anemia, of whom 36.2% (n=161) were anemic. Eighty-two out of the 561 had undergone HPLC testing, and 38 (5.9%) were confirmed as thalassemia trait. Only 32 out of the confirmed thalassemia trait participants were anemic during the first-trimester assessment. A quarter (n=40) of anemic pregnant women with a history of anemia had a high Red Cell Index suggestive of minor hemoglobinopathy. Among the anemic pregnant women, one-third of ethnic Moore/Malay women (n=19) had a high red cell index compared to only 18.2% (n=72) of ethnic Sinhalese/Tamil women. Respectively 198, 360, 39, and 52 women with anemia had received dietary advice, oral iron, intravenous iron, and other vitamins as treatment for anemia at the time of the previous diagnosis. 17 had received blood transfusions of which 11 were during the antenatal period of a previous pregnancy. Only 156 had evidence of normalized hemoglobin levels after initial treatments. [u1]

Chi-square tests showed that the [u2] anemia prevalence was statistically significantly different between groups categorized according to the gap between current and last pregnancy, body mass index (BMI) category, history of anemia, having a vehicle at home, having used intrauterine contraceptive device (IUCD) for one year or more, having used depo-medroxyprogesterone acetate injection (DMPA) for a year or more and breastfeeding during the past year (Table 1).

**Table 1 Factors associated with anemia among first-trimester pregnant women in Anuradhapura district**

|  |  | **Anaemic** | | **Non-anaemic** | |  | |
| --- | --- | --- | --- | --- | --- | --- | --- |
|  |  | N | % | N | % | Χ^2^ | p |
| **Demographic factors** | | | |  |  |  |  |
| Age | <20 | 36 | 7.9 | 169 | 6.3 |  |  |
|  | 20-24 | 87 | 19.0 | 528 | 19.7 |  |  |
|  | 25-29 | 161 | 35.2 | 931 | 34.8 | 6.99 | 0.22 |
|  | 30-34 | 102 | 22.3 | 688 | 25.7 |  |  |
|  | 35-39 | 56 | 12.3 | 312 | 11.6 |  |  |
|  | ≤40 | 15 | 3.3 | 51 | 1.9 |  |  |
| Education level | Up to O/L | 274 | 61.3 | 1587 | 59.9 | 0.31 | 0.58 |
|  | Beyond O/L | 173 | 38.7 | 1062 | 40.1 |  |  |
| Partner's education level | Up to O/L | 307 | 68.2 | 1800 | 68.2 | 0.00 | 0.99 |
|  | Beyond O/L | 143 | 31.8 | 840 | 31.8 |  |  |
| Family size | <5 | 294 | 65.6 | 1811 | 68.3 | 1.30 | 0.25 |
|  | ≥5 | 154 | 34.4 | 839 | 31.7 |  |  |
| Ethnicity | Sinhala/Tamil | 397 | 87.3 | 2356 | 88.1 | 0.27 | 0.60 |
|  | Moore/Malay | 58 | 12.7 | 318 | 11.9 |  |  |
| **Factors related to fertility and reproduction** | | | | | | | |
| Number of children | Non | 143 | 31.2 | 817 | 30.5 | 0.34 | 0.84 |
|  | One | 147 | 32.5 | 849 | 31.8 |  |  |
|  | Two or more | 164 | 36.3 | 1007 | 37.7 |  |  |
| Planned pregnancy | Yes | 326 | 72.3 | 1950 | 73.9 | 0.55 | 0.46 |
|  | No | 125 | 27.7 | 687 | 26.1 |  |  |
| The gap between current and last pregnancy | <5 years | 147 | 32.7 | 1084 | 40.8 | 13.29 | 0.001 |
|  | ≥5 years | 160 | 35.6 | 754 | 28.4 |  |  |
|  | Primiparous | 143 | 31.8 | 817 | 30.8 |  |  |
| Breast feeding currently/within last one year | No | 301 | 69.8 | 1590 | 62.4 | 8.85 | 0.003 |
|  | Yes | 130 | 30.2 | 959 | 37.6 |  |  |
| Have had miscarriages ever | No | 230 | 74.4 | 1343 | 72.8 | 0.36 | 0.55 |
|  | Yes | 79 | 25.6 | 502 | 27.2 |  |  |
| Has regular menstrual cycles | Yes | 388 | 85.5 | 2229 | 83.5 | 1.12 | 0.29 |
|  | No | 66 | 14.5 | 441 | 16.5 |  |  |
| Average menstrual blood loss (before pregnancy) | Low | 138 | 32.5 | 865 | 33.7 | 0.96 | 0.62 |
|  | Moderate | 138 | 32.5 | 864 | 33.7 |  |  |
|  | High | 149 | 35.1 | 838 | 32.6 |  |  |
| Has used injectable contraceptives for ≥1year | No | 369 | 80.7 | 1997 | 74.5 | 8.17 | 0.004 |
|  | Yes | 88 | 19.3 | 683 | 25.5 |  |  |
| Has used intrauterine contraceptive devices for ≥1year | No | 389 | 85.1 | 2422 | 90.4 | 11.57 | 0.001 |
|  | Yes | 68 | 14.9 | 258 | 9.6 |  |  |
| **Current pregnancy-related factors** | | | | | | | |
| Attended pre pregnancy sessions | Yes | 71 | 22.5 | 431 | 23.1 | 0.05 | 0.82 |
|  | No | 244 | 77.5 | 1434 | 76.9 |  |  |
| Had blood in stools within last 6 months | Yes | 15 | 3.4 | 111 | 4.2 | 0.64 | 0.42 |
|  | No | 428 | 96.6 | 2534 | 95.8 |  |  |
| Had melena within last 6 months | Yes | 9 | 2.0 | 45 | 1.7 | 0.22 | 0.64 |
|  | No | 438 | 98.0 | 2600 | 98.3 |  |  |
| Had taken anthelminthic drugs within last 6 months | Yes | 195 | 43.6 | 1151 | 43.5 | 0.00 | 0.96 |
|  | No | 252 | 56.4 | 1495 | 56.5 |  |  |
| **Diet and nutritional supplements** | | | | | | | |
| Frequency of consuming meat/fish | Low | 64 | 35.2 | 466 | 42.4 | 3.38 | 0.18 |
|  | Moderate | 67 | 36.8 | 366 | 33.3 |  |  |
|  | High | 51 | 28.0 | 268 | 24.4 |  |  |
| Frequency of consuming milk/dairy products | Low | 85 | 46.7 | 378 | 34.5 | 10.27 | 0.006 |
|  | Moderate | 59 | 32.4 | 452 | 41.2 |  |  |
|  | High | 38 | 20.9 | 267 | 24.3 |  |  |
| Has consumed iron supplements during last pregnancy | Yes | 269 | 91.5 | 1625 | 92.9 | 0.69 | 0.41 |
|  | No | 25 | 8.5 | 125 | 7.1 |  |  |
| Has consumed iron supplements during postpartum period of last pregnancy | Yes, regularly | 178 | 61.6 | 1067 | 61.6 | 0.00 | 1.00 |
|  | Yes, not regularly | 28 | 9.7 | 168 | 9.7 |  |  |
|  | No | 83 | 28.7 | 496 | 28.7 |  |  |
| Consumed folic acid supplements before pregnancy | Yes | 266 | 81.8 | 1559 | 80.4 | 0.37 | 0.54 |
|  | No | 59 | 18.2 | 380 | 19.6 |  |  |
| Consuming folic acid supplements currently | Yes | 421 | 93.3 | 2521 | 94.7 | 1.36 | 0.24 |
|  | No | 30 | 6.7 | 141 | 5.3 |  |  |
| **Nutritional status** |  |  |  |  |  |  |  |
| History of anaemia | No | 284 | 63.8 | 2210 | 83.9 | 104.52 | 0.001 |
|  | Yes, during a past pregnancy/postpartum | 109 | 24.5 | 319 | 12.1 |  |  |
|  | Yes, not during a past pregnancy | 52 | 11.7 | 106 | 4.0 |  |  |
| Waist to hip ratio | ≥88 cm | 282 | 66.2 | 1707 | 67.4 | 0.25 | 0.62 |
|  | <88 cm | 144 | 33.8 | 825 | 32.6 |  |  |
| BMI | Underweight | 101 | 23.0 | 403 | 15.5 | 20.22 | 0.001 |
|  | Normal | 152 | 34.6 | 841 | 32.4 |  |  |
|  | Overweight/obese | 186 | 42.4 | 1352 | 52.1 |  |  |
| **Socioeconomic conditions** | | | | | | | |
| Have/had the habit of smoking tobacco products | Yes | 2 | 0.5 | 7 | 0.3 | 0.43 | 0.512 |
|  | No | 398 | 99.5 | 2347 | 99.7 |  |  |
| Have/ Had the habit of betel chewing (ever) | Yes | 13 | 3.2 | 43 | 1.8 | 3.46 | 0.06 |
|  | No | 388 | 96.8 | 2313 | 98.2 |  |  |
| Has someone who smokes at home | Yes | 79 | 19.8 | 413 | 17.8 | 0.91 | 0.34 |
|  | No | 321 | 80.3 | 1912 | 82.2 |  |  |
| Have/ever had the habit of consuming alcoholic beverages | Yes | 3 | 0.8 | 17 | 0.8 | 0.01 | 0.920 |
|  | No | 362 | 99.2 | 2185 | 99.2 |  |  |
| Have been emotionally or physically abused ever | Yes | 18 | 4.5 | 80 | 3.4 | 1.35 | 0.25 |
|  | No | 379 | 95.5 | 2293 | 96.6 |  |  |
| Uses a water sealed toilet | Yes | 401 | 89.5 | 2438 | 92.1 | 3.30 | 0.07 |
|  | No | 47 | 10.5 | 210 | 7.9 |  |  |
| Has electricity at home | Yes | 435 | 97.3 | 2605 | 98.4 | 2.69 | 0.10 |
|  | No | 12 | 2.7 | 42 | 1.6 |  |  |
| Has a mobile phone | Yes | 438 | 97.8 | 2613 | 98.7 | 2.46 | 0.12 |
|  | No | 10 | 2.2 | 34 | 1.3 |  |  |
| Uses biomass fuel for cooking | Yes | 201 | 44.9 | 1314 | 49.6 | 3.44 | 0.06 |
|  | No | 247 | 55.1 | 1335 | 50.4 |  |  |
| Drinks water filtered using Reverse Osmosis | Yes | 312 | 69.8 | 1872 | 70.7 | 0.14 | 0.71 |
|  | No | 135 | 30.2 | 777 | 29.3 |  |  |
| Has a vehicle at home | Yes | 391 | 89.1 | 2401 | 92.0 | 4.28 | 0.038 |
|  | No | 48 | 10.9 | 208 | 8.0 |  |  |
| Monthly family income category | 1 (Lowest) | 50 | 28.4 | 280 | 26.4 | 1.55 | 0.67 |
|  | 2 | 37 | 21.0 | 263 | 24.8 |  |  |
|  | 3 | 41 | 23.3 | 255 | 24.1 |  |  |
|  | 4(Highest) | 48 | 27.3 | 262 | 24.7 |  |  |

Hierarchical logistic regression was performed to assess the impact of several factors on the likelihood of anemia in the first trimester of pregnancy (Table 2). The PoG in weeks, maternal age (years) and parity (primy or multi) were added in block one. Having been breastfeeding within the past one year, ever having had anemia, BMI (reference category was those who were not underweight), having a vehicle at home, the gap between last and current pregnancies (reference category was being a primy or having the last pregnancy within five years), having used contraceptive injections for one year or more and having used intrauterine contraceptive devices for one year or more were added to the model as the block two. The final model was statistically significant (Chi-square =139.3, p<0.001, n=2692), which could explain 5.0% (Cox and Snell R square) and 9.2% (Nagaelkerke R squared) of the variance and correctly classified 86.2% of cases. The greatest contribution to the model was from having a history of anemia. Those who had a history of anemia were 3.2 (P<0.001) times more likely to have anemia during the first trimester of pregnancy. Odds of having anemia increased with increasing PoG. Using intrauterine contraceptive devices for one year or more also increased the odds of having anemia by 1.6 times compared to those who had not used IUCD for a year or more. Having used DMPA injections for one year or more was protective against anemia. Those who were breastfeeding or had breastfed within the past year were less likely to be anemic than those who had never breastfed/had stopped breastfeeding more than a year ago. Underweight pregnant women were 1.6 times more likely to be anemic than pregnant women with normal BMI, overweight or obese. Pregnant women who had had their last pregnancy five or more years back were 1.6 times more likely to have anemia compared to primiparous women and those who had their last pregnancy within five years.

**Table 2 Higherachial logistic regression explains anemia in first trimester pregnant women in Anuradhapura district.**

|  | B | S.E. | Sig. | Odds ratio | 95% CI for OR | |
| --- | --- | --- | --- | --- | --- | --- |
|  |  |  |  |  | Lower | Upper |
| Period of gestation (weeks) | 0.07 | 0.03 | 0.018 | 1.07 | 1.01 | 1.13 |
| Maternal age (years) | -0.01 | 0.01 | 0.408 | 0.41 | 0.97 | 1.02 |
| Parity (primy or multi) | -0.06 | 0.20 | 0.782 | 0.95 | 0.64 | 1.41 |
| Having breast fed within one year | -0.41 | 0.16 | 0.009 | 0.66 | 0.49 | 0.90 |
| History of anaemia | 1.17 | 0.13 | 0.001 | 3.22 | 2.51 | 4.13 |
| Underweight (reference group- Normal/ overweight/ obese) | 0.5 | 0.15 | 0.001 | 1.64 | 1.24 | 2.18 |
| No vehicle at home | 0.32 | 0.19 | 0.099 | 1.37 | 0.95 | 2.00 |
| Used DMPA one year or more | -0.49 | 0.18 | 0.002 | 0.61 | 0.45 | 0.83 |
| Used IUCD one year or more | 0.49 | 0.18 | 0.005 | 1.63 | 1.16 | 2.30 |
| Last pregnancy 5 or more years back (reference group – primy/ last pregnancy within 5 years) | 0.45 | 0.16 | 0.005 | 1.57 | 1.15 | 2.15 |

**Discussion**

This large community-based study provides a detailed analysis of probable associations of early pregnancy anemia, including demographic, nutritional, reproductive and socioeconomic factors. This study has shown that having a history of anemia, being underweight, having the last pregnancy five or more years back, and having used IUCD for a significant period were associated with higher odds of being anemic. Having been breastfeeding during the last year and having used DMPA injections for contraception were associated with lower odds of being anemic. The odds of being anemic also increased with the period of gestation at which the hemoglobin was tested. The study reflects that anemia during the first trimester of pregnancy represents the impact of pre-pregnancy determinants, including lifestyle, nutrition and reproductive choices, on the health of pregnant women. While identifying the etiologies is essential, strengthening the lifecycle approach in providing care for reproductive and other health issues would successfully address almost all the factors associated with early pregnancy anemia in this community.

We could observe that anemia increased with PoG (OR=1.07, 95% CI 1.01 to 1.13). This observation can at least partly be explained through the hemodilution of pregnancy. Looking at this pattern, we could argue that using 11g/dl as the hemoglobin cutoff until 13 weeks of PoG may classify some pregnant women who have lower hemoglobin levels purely due to physiological changes in pregnancy as anemic. Such mothers will undergo unnecessary interventions such as additional investigations, increased iron dose and other nutritional supplements and dietary modifications that may lead to increased psychological and economic burden. Therefore, the harms and benefits of redefining anemia cutoff for early pregnancy should be studied further through prospective studies.

Controlling for PoG, maternal age and parity, the strongest predictor of first-trimester anemia, according to this study, was having had anemia earlier in life. Non-modifiable etiologies such as thalassemia trait and membrane disorders contribute to a significant proportion of anemia in Sri Lankan communities (18,19). For example, a quarter of anemic women with a history of anemia in this study sample had minor haemoglobinopathies according to their RBC index. On the other hand, treating anemia without a proper follow-up and etiological diagnosis may also lead to unresolved or recurrent anemia in women which may be carried on to the pregnancy. More than two-thirds of the women with a history of anemia was diagnosed during a previous pregnancy. Hence paying attention to ascertain the etiology for anaema identified in pregnant women and following them up during postnatal period would be beneficial in view of continuum of care.

Compared to primiparous women and women who had a pregnancy within the last five years, having had the last pregnancy five or more years back was associated with an increased risk for anemia. A study among non-pregnant women has also shown a similar trend of increased prevalence of anemia with the increased pregnancy gap(20). Similarly, having breastfed a baby within the last year is also protective of anemia after controlling for age and pregnancy gap. These findings contrast with previous global literature showing that breastfeeding and lower pregnancy gaps increase the risk for anemia in women (3,4). A possible contextual explanation for this would be that resent contact with the public health system could be beneficial for maintaining the nutritional status of women in this community. Newly married women can attend the preconsceptional clinics and receive nutritional supplementations and advice. Nutritional supplements including iron-folate and ‘Thriposha’ are continued from antenatal period to six months postpartum. Having a child under five years of age also brings the family to close contact with the public health system. Attention given to women and children during the first few years of delivery through domiciliary and clinic care provided by the primary health care personnel is high in Sri Lanka. In the context where interpregnancy care is not strong or well structured, women who do not have a frequent contact with the public health system may be at a disadvantage (6).

Using DMPA and IUCD for a significant period decreases and increases the risk of anemia, respectively. Copper IUCD provided by the national family planning programme is the commonly used type of IUCD which is known to increase menstrual blood loss and reduce iron stores in women (21,22). This may predispose women to develop anemia in the first trimester of pregnancy, especially when it occurs on top of already depleted nutritional stores or minor haemoglobinopathy variants. As IUCD is considered an ethnic appropriate modern method due to the absence of hormonal effects, it would be valuable to investigate the above observation further. On the other hand, DMPA can cause temporary cessation of menstruation in many users (23). Reduced menstrual loss may allow women to maintain their hemoglobin and iron levels. Providing adequate follow up for contraceptive users and prompt treatment of excessive menstrual bleeding associated with contraceptive methods could help reduce anemia in reproductive-age females leading to reduction of anemia in early pregnancy.

Comparable to global evidence (3), underweight pregnant women had 1.64 (95% CI 1.24	- 2.18) times more risk of being anemic than pregnant women with normal BMI. This signifies the association between anemia and nutrition during pre-pregnancy periods. A study among primary school children from the same setting has also shown an association between BMI and anemia, indicating that anemia and macro-nutrient deficiencies may be linked through common causative factors (24). However, the income level or assets such as vehicles were not statistically significantly associated with anemia in the current study. Most of the dietary behaviors such as consumption of meat, fish, eggs or green leaves, which have shown to be associated with reducing the risk for anemia are also not significantly associated in this community. Only the group whose milk and dairy products consumption was low included a comparatively higher percentage of women with anemia than the group reporting moderate or high consumption levels. While this observation may be directly related to the nutrient intake from the dietary pattern it may also indirectly reflect the socioeconomic aspects such as the pattern of expenditure on food items.

The study was conducted in a rural district of the country. The study's internal validity is high as the total study population was invited to participate, and the participation rate was high (86%). However, inter-district variations in the epidemiological patterns of anaemia should be considered when generalizing the results to the country or beyond.

**Conclusion**

Looking at the reproductive and nutrition-related associations to early pregnancy anemia in the current study results, we hypothesize that being in frequent contact with the national family health programme succeeds over socioeconomic disparities in preventing anemia in early pregnancy. Therefore, quantitative and qualitative improvement of already implemented nutritional interventions is important, and attention should be paid to maintaining them even during crises like the SARS Covid-19 pandemic. Even though the national programme focuses on a continuum of care across the life cycle, interpregnancy care and care for eligible couples are still not strong. Strategies to mend this gap will reduce the burden of anemia in pregnancy through assessment and optimization of the macro and micronutrient status of women prior to pregnancy.

- Acknowledgments
- Supporting information captions )
- Abbreviations

**Reference**

1.	WHO. Anaemia [Internet]. World Health Organization. 2015. Available from: http://www.who.int/topics/anaemia/en/

2.	Anaemia [Internet]. [cited 2021 Dec 20]. Available from: https://www.who.int/health-topics/anaemia#tab=tab_1

3.	Owais A, Merritt C, Lee C, Bhutta ZA. Anemia among Women of Reproductive Age: An Overview of Global Burden, Trends, Determinants, and Drivers of Progress in Low-and Middle-Income Countries. 2021 [cited 2021 Dec 20]; Available from: https://doi.org/10.3390/nu13082745

4.	Ali SA, Khan U, Feroz A. Prevalence and Determinants of Anemia among Women of Reproductive Age in Developing Countries. Journal of the College of Physicians and Surgeons--Pakistan : JCPSP [Internet]. 2020 Feb 1 [cited 2021 Dec 20];30(2):177–86. Available from: https://pubmed.ncbi.nlm.nih.gov/32036827/

5.	Family Health Bureau SL. Maternal Care Package A Guide to Field Healthcare Workers. Family Health Bureau Ministry of Health. 2011.

6.	Family Health Burro Sri Lanka. Annual Report_2019 [Internet]. Colombo; 2018 [cited 2022 Jan 8]. Available from: https://drive.google.com/file/d/1EsRRNgINb5uUxJMQLnwX3TfoAHa1Nc6q/view

7.	Mudalige R, Nestel P. Combating Iron Deficiency 2. Prevalence of Anaemia in Sri Lanka. The Ceylon Journal of Medical Science. 1996;39:9–16.

8.	Jayatissa R, Fernando DN, de Silva H. National Nutrition and Micronutrient Survey of Pregnant Women in Sri Lanka 2015. Colombo; 2017.

9.	Atukorala TMS, de Silva LDR, Dechering WHJC, de C Dassenaeike TS, Perera RS. Evaluation of effectiveness of iron-folate supplementation and anthelminthic therapy against anemia in pregnancy - A study in the plantation sector of Sri Lanka. American Journal of Clinical Nutrition. 1994;60(2):286–92.

10.	Kandauda IC, Tennakoon S, Rathnayake PM, Maduwage K, Gunathilake T. Prevalence and aetiology of anaemia in pregnant women attending a tertiary care unit in sri lanka, and its effect on the mother and newborn. Sri Lanka Journal of Medicine [Internet]. 2020 Dec 15 [cited 2021 Dec 20];29(2):37. Available from: http://sljm.sljol.info/articles/10.4038/sljm.v29i2.223/

11.	Chathuranga G, Balasuriya T, Perera R. Anaemia among Female Undergraduates Residing in the Hostels of University of Sri Jayewardenepura, Sri Lanka. 2014 [cited 2021 Dec 4]; Available from: http://dx.doi.org/10.1155/2014/526308

12.	Department of Census and Statistics, Department of Census and Statistics Sri Lanka. Prevalence of Anaemia among Children and Women Demographic and Health Survey 2006/7. Health Sector Development Project Ministry of Healthcare and Nutrition; 2006.

13.	Goonewardene IMR, Deeyagaha Waduge RPK. Adverse effects of teenage pregnancy. The Ceylon medical journal [Internet]. 2005 Sep [cited 2016 Dec 18];50(3):116–20. Available from: http://www.ncbi.nlm.nih.gov/pubmed/16252576

14.	Agampodi TC, Wickramasinghe ND, Indika R, Prasanna R, Mudiyanselage P, Jayathilake B, et al. The Rajarata Pregnancy Cohort ( RaPCo ): study protocol. BMC pregnancy and childbirth. 2020;20(374):1–13.

15.	Amarasinghe GS, Agampodi TC, Mendis V, Malawanage K, Kappagoda C, Agampodi SB. Prevalence and aetiologies of anaemia among first trimester pregnant women in Sri Lanka; the need for revisiting the current control strategies. BMC Pregnancy and Childbirth 2022 22:1 [Internet]. 2022 Jan 6 [cited 2022 Jan 8];22(1):1–12. Available from: https://bmcpregnancychildbirth.biomedcentral.com/articles/10.1186/s12884-021-04341-z

16.	Agampodi SB, Amarasinghe G. Anemia Tools [Internet]. OSF; 2020 [cited 2022 Jan 4]. Available from: https://osf.io/ksx2f/

17.	Amarasinghe G, Mendis V, Agampodi T. Cross-sectional study to assess etiology and associated factors for anaemia during first trimester of pregnancy in Anuradhapura District, Sri Lanka: a protocol. F1000Research [Internet]. 2021 Mar 18 [cited 2021 Apr 9];10:223. Available from: https://doi.org/10.12688/f1000research.28226.1

18.	Mettananda S, Paranamana S, Fernando R, Suranjan M, Rodrigo R, Perera L, et al. Microcytic anemia in children: parallel screening for iron deficiency and thalassemia provides a useful opportunity for thalassemia prevention in low- and middle-income countries. Pediatric Hematology and Oncology. 2020 May 18;37(4):326–36.

19.	Warushahennad R, Wanasundara DH, Priyadarshani W, Abeysekara AM, Jiffry MN. Prevalence of β thalassaemia trait and Haemoglobin E disorders among students aged between 14-17yrs in Kurunegala district, Sri Lanka. Ceylon Medical Journal [Internet]. 2020 Jun 30 [cited 2021 Dec 4];65(1–2):9. Available from: http://cmj.sljol.info/articles/10.4038/cmj.v65i1-2.9131/

20.	Sheriff FA, Dissanayake RK, Wijesundara C, Bandaranayaka KO, Chathuranga G. Prevalence, knowledge and selected associated factors of anaemia among non-pregnant females of reproductive age in a tea estate community in Hantana, Kandy district, Sri Lanka. American Journal of Blood Research [Internet]. 2021 [cited 2021 Dec 4];11(4):438. Available from: /pmc/articles/PMC8446824/

21.	Sivin I, Stern J. Health during prolonged use of levonorgestrel 20 micrograms/d and the copper TCu 380Ag intrauterine contraceptive devices: a multicenter study. International Committee for Contraception Research (ICCR). Fertility and sterility [Internet]. 1994 [cited 2021 Dec 10];61(1):70–7. Available from: https://pubmed.ncbi.nlm.nih.gov/8293847/

22.	Andrade ATL, Orchard EP. Quantitative studies on menstrual blood loss in IUD users. Contraception [Internet]. 1987 [cited 2021 Dec 10];36(1):129–44. Available from: https://pubmed.ncbi.nlm.nih.gov/3311622/

23.	World Health Organization, Johns Hopkins Bloomsberg School of Public Health, United States Agency for International Development. Family Planning - A Global Handbook for Providers [Internet]. 3rd ed. Geneva: WHO; 2018 [cited 2022 Jan 4]. 97–118. Available from: http://apps.who.int/iris/bitstream/handle/10665/260156/9780999203705-eng.pdf;jsessionid=92DAE99D14DD392367E5535FCA511A67?sequence=1

24.	Amarasinghe GS, Naottunna NPGCR, Agampodi TC, Agampodi SB. Factors associated with anemia among Sri Lankan primary school children in rural North Central Province. BMC Pediatrics. 2017 Mar 27;17(1).
